# Circulating microRNA profiles and the identification of miR-593 and miR-511 which directly target the *PROP1* gene in children with combined pituitary hormone deficiency

**DOI:** 10.3892/ijmm.2014.2016

**Published:** 2014-11-28

**Authors:** YANYAN HU, QIAN WANG, ZENGMIN WANG, FENGXUE WANG, XIAOBO GUO, GUIMEI LI

**Affiliations:** 1 Departments of Pediatrics, Shandong Provincial Hospital Affiliated to Shandong University, Jinan, Shandong 250021, P.R. China; 2 Gastrointestinal Surgery, Shandong Provincial Hospital Affiliated to Shandong University, Jinan, Shandong 250021, P.R. China

**Keywords:** microRNAs, *PROP1* gene, combined pituitary hormone deficiency, microarray

## Abstract

Since the tissue of children with combined pituitary hormone deficiency (CPHD) is not readily accessible, a new focus in children with CPHD is the blood-based expression profiling of non-protein coding genes, such as microRNAs (miRNAs or miRs), which regulate gene expression by inhibiting the translation of mRNAs. In this study, to address this, we identified potential miRNA signatures for CPHD by comparing genome-wide miRNA expression profiles in the serum of children with CPHD vs. normal (healthy) controls. Human embryonic kidney 293T cells were transfected with miR-593 or miR-511 oligonucleotides. Potential target gene expression was validated by western blot analysis for proteins and by miR-593 or miR-511 reporter assay using *PROP1* gene 3′-untranslated region (3′-UTR) reporter. The miR-593 and miR-511 levels in the serum of 103 children with CPHD were assessed using the reverse transcription-quantitative polymerase chain reaction (RT-qPCR) method. We found 23 upregulated and 19 down-regulated miRNAs with abnormal expression in children with CPHD compared with the normal controls using miRNA microarray analysis and RT-qPCR. miR-593 and miR-511 targeted the 3′-UTR of the *PROP1* gene and attenuated the expression of *PROP1*. The levels of miR-593 and miR-511 in the serum of children with CPHD were increased compared with those in the control subjects. According to Youden’s index, the sensitivity was 82.54 and 84.86%, and the specificity was 98.15 and 91.36% for miR-593 and miR-511, respectively. The various levels of specific miRNAs, particularly miR-593 and miR-511 whose direct target is the *PROP1* gene, may serve as a non-invasive diagnostic biomarkers for children with CPHD.

## Introduction

Great progress has been made in our understanding of the development of the anterior pituitary gland and of the mechanisms that underlie the diagnosis of combined pituitary hormone deficiency (CPHD). Naturally occurring and transgenic murine models have demonstrated a role for many of these molecules in the etiology of CPHD (1,2). Anatomical abnormalities in the pituitary gland may be associated with other midline abnormalities and variable endocrine disorders, ranging from isolated growth hormone deficiency (IGHD) to CPHD (3,4). CPHD is a severe endocrine disorder in children. Different types and severities of hormonal deficiencies with various clinical manifestations are observed in children with CPHD. The definite diagnosis of CPHD is necessary. Pituitary magnetic resonance imaging (MRI) and hormones are essential examinations for the diagnosis of CPHD. Significant advances in molecular biology and the normal development of the pituitary gland have led to a greater understanding of the genetic basis of CPHD and related conditions.

*PROP1* has been mapped to chromosome 5q and encodes a protein of 226 amino acids. The DNA-binding homeodomain consists of 3 α-helical regions and the majority of mutations reported to date affect this region. *PROP1* is essential for the differentiation of gonadotrophs in fetal life. The spectrum of gonadotropin deficiency is again extremely variable, ranging from hypogonadism and the lack of puberty to spontaneous pubertal development and infertility (5,6). However, it is has been suggested that *PROP1* is not required for gonadotroph determination, but is required for differentiation. A 2-bp deletion (delA301, G302) is now believed to be a mutational ‘hot spot’ within *PROP1* (7–9). To date, mutations in *PROP1* are associated with growth hormone (GH), thyrotropin (TSH), prolactin (PRL) and gonadotropin deficiencies. Fifteen distinct recessive mutations have been identified in approximately 147 individuals from 76 to 84 pedigrees originating in 20 different countries, suggesting that mutations within *PROP1* are the most common genetic cause of CPHD, with incidence rates quoted between 50 and 100% in familial cases of CPHD (10–12).

Recently, researchers have found a new class of short, endogenously non-coding RNAs termed microRNAs (miRNAs or miRs) in animals and plants (13–15). It is now clear that they play pivotal roles in a wide array of biological processes, including differentiation and cell proliferation and apoptosis (16,17). They regulate the expression of protein-coding genes by degrading or inhibiting the translation of the targeted mRNAs (18). Emerging evidence strongly suggests that abnormal miRNA expression is a common and important characteristic of human diseases (19,20). To date, a number of studies have proven that a non-invasive approach for the circulating blood-based miRNA identification of biomarkers is extremely valuable and useful in diseases (17,19–21).

miRNA profiling using microarray technology has recently been developed and applied to the study of a variety of conditions (22,23). Based on these studies, we can now perform blood-based miRNA profiling to search for CPHD. In this study, to ascertain whether circulating miRNA expression signatures can distinguish children with CPHD from normal (healthy) controls, we performed genome-wide miRNA expression profiling from serum samples in children with CPHD and healthy controls. Using expression profile data and data from reverse transcription-quantitative PCR (RT-qPCR), our study indicates that the various levels of specific miRNAs, particularly miR-593 and miR-511 whose direct target is the *PROP1* gene, may serve as non-invasive diagnostic biomarkers for children with CPHD.

## Materials and methods

### Blood sample collection

A total of 206 participants at the Department of Pediatrics of Shandong Provincial Hospital Affiliated to Shandong University (Jinan, China) between 2009 and 2013 were recruited in this study. This included 103 children with CPHD (88 boys and 15 girls; age, 11.6±3.5 years; range, 8.2–16.6 years) and 103 normal (healthy) controls (85 boys and 18 girls; age, 11.2±3.8 years; range, 7.5–16.0 years). There were no significant differences in the age and gender between the CPHD group and the control group (P>0.05). All children had at least one anterior pituitary hormone deficiency in addition to GHD, and were therefore diagnosed as having CPHD. Whole blood samples (4 ml) were collected from the children with CPHD and the normal controls into K2-EDTA-coated tubes. Subsequently, 3 ml of the whole blood was centrifuged at 1,000 × g for 10 min, and then the serum (approximately 1 ml) was aliquoted into an RNase-free tube. All the serum samples were stored at -80°C prior to RNA extraction. The study was approved by the institutional review board of the hospital. Written informed consent was obtained from the parents of all the subjects.

### miRNA microarray

Total RNA from 7 children with CPHD and 7 normal controls was isolated using TRIzol reagent (Invitrogen, Carlsbad, CA, USA) and the miRNeasy Mini kit (Qiagen, Hilden, Germany) according to manufacturer’s instructions. For each sample that passed RNA quantity measurement using the NanoDrop 1000 spectrophotometer (Thermo Fisher Scientific, Waltham, MA, USA) 1 *μ*g of total RNA was 3′-end-labeled with Hy3™ fluorescent label using the miRCURY™ Hy3™/Hy5™ Power Labeling kit (Exiqon, Vedbaek, Denmark), and hybridized to miRCURY^™^ LNA Arrays (version 18.0), according to the manufacturer’s instructions. The seventh generation of miRCURY^™^ LNA Arrays (version 18.0) (Exiqon) contains 3,100 capture probes, covering all human, mouse and rat miRNAs annotated in miRBase 18.0. In addition, this array contains capture probes for 25 miRPlus™ human miRNAs. Following hybridization, the slides were washed several times using the Wash buffer kit (Exiqon), and dried by centrifugation for 5 min at 400 rpm. The slides were scanned using the Axon GenePix 4000B microarray scanner (Axon Instruments, Foster City, CA, USA). Scanned images were imported into GenePix Pro 6.0 software (Axon Instruments) for grid alignment and data extraction. The data were normalized using median normalization. Following normalization, differentially expressed miRNAs were identified through fold change filtering. Hierarchical clustering was performed using MEV software (version 4.6, TIGR).

### Bioinformatics: sequence analysis

We researched microRNAs that were associated with children with CPHD through the miRBase (http://microrna.sanger.ac.uk/). Putative targets were identified using the microrna.org (http://www.microrna.org/microrna/home.do), TargetScan (http://www.targetscan.org/vert_40/) and RNAhybrid databases (http://bibiserv.techfak.uni-bielefeld.de/rnahybrid/submission.html).

### Cell lines, culture and transfection

The human embryonic kidney (HEK)293T cells were preserved at our institute and cultured in Dulbecco’s modified Eagle’s medium (DMEM; Sigma, St. Louis, MO, USA) supplemented with 10% fetal calf serum (FCS). Exponentially growing cells were used for the experiments.

Stability-enhanced miRNAs, stability-inhibited miRNAs and negative control RNA-oligonucleotides were obtained from Ambion Inc. (Austin, TX, USA). The day prior to transfection, the HEK293T cells were seeded in antibiotic-free medium. The transfection of miRNAs was carried out using Lipofectamine 2000 in accordance with the manufacturer’s instructions (Invitrogen). Stability-enhanced miRNAs, stability-inhibited miRNAs and negative control RNA-oligonucleotides were transfected at a final concentration of 50 nM unless otherwise indicated. The level of miR-593 and miR-511 expression in the transfected HEK293T cells was assayed by RT-qPCR 48 h after transfection as described below.

### RT-qPCR

Total RNA from was extracted from the serum samples and cultured cells using a miRNeasy Mini kit (Qiagen) designed to isolate small molecular weight nucleic acids. The concentration and purity of the total RNA samples were measured using the SmartSpec Plus spectrophotometer (Bio-Rad, Hercules, CA, USA). The ratio of A260:A280 was used to indicate the purity of total RNA.

cDNA was generated using the miScript Reverse Transcription (RT) kit (Qiagen). According to the manufacturer’s instructions, 1 *μ*g total RNA, 1 *μ*l miScript reverse transcriptase mix, 4 *μ*l 5X miScript RT buffer and appropriate volume RNase-free water were mixed well and incubated for 60 min at 37°C, and then incubated for 5 min at 95°C to inactivate miScript reverse transcriptase mix. All reverse transcription procedures and no-template controls were run at the same time.

A miScript SYBR-Green PCR kit (Qiagen) was used to measure the expression of mature miR-593 and miR-511 in the samples and cells following reverse transcription. Quantitative (real-time) PCR was performed using on an Mx3005P qPCR System (Stratagene, La Jolla, CA, USA). Following the manufacturer’s instructions, the 20 *μ*l PCR mixture included 2 *μ*l reverse transcription product, 10 *μ*l 2X QuantiTect SYBR-Green PCR Master Mix, 2 *μ*l 10X miScript Universal Primer, 2 *μ*l 10X miScript Primer Assay (for miR-593 and miR-511; Qiagen) and 4 *μ*l RNase-free water. The reaction mixtures were incubated at 95°C for 15 min, followed by 40 amplification cycles of 94°C for 15 sec, 55°C for 30 sec and 70°C for 30 sec. We also quantified transcripts of U6 small nuclear RNA using the Hs_RNU6B_2 miScript Primer Assay (Qiagen) for normalizing the levels of miR-593 and miR-511. Hs_RNU6B_2 was used as an endogenous control. Each sample was analyzed 2 times. The threshold cycle (Ct) was defined as the fractional cycle number at which the fluorescence exceeds the given threshold. The obtained data were translated into the log2 scale, as previously described (29). The 2^-ΔΔCt^ method was used to analyze the relative expression of the miRNAs.

### Western blot analysis

The HEK293T cells were transfected with miR-593 precursor, miR-511 precursor, miR-593 inhibitor, miR-511 inhibitor, or the negative control in 6-well plates. Following transfection, the cells were cultured for 48 h, protein was extracted using mammalian protein extraction reagent (Pierce, Rockford, IL, USA) supplemented with protease inhibitors cocktail (Sigma). Protein samples (50 *μ*g) were resolved by 10% sodium dodecyl sulfate-polyacrylamide gel electrophoresis (SDS-PAGE) and then transferred onto PVDF membranes. The membranes were blocked with TBST buffer (TBS plus 0.1% Tween-20) containing 5% w/v non-fat milk and hybridized with primary antibody (rabbit polyclonal antibody, ab94500), followed by incubation with specific HRP-conjugated secondary antibody (anti-rabbit IgG antibody, ab191866). Protein bands were visualized using the ECL detecting system (Amersham Biosciences, Uppsala, Sweden). Rabbit polyclonal anti-PROP1 (1:1,000; ab94500; Abcam, Cambridge, MA, USA) and anti-rabbit IgG antibody (1:1,000; ab191866; Abcam) were used. Monoclonal anti-GAPDH (1:5,000; ab181602; Abcam) was used as a loading control.

### Luciferase activity assay

A 230-bp fragment of the wild-type *PROP1* 3′-untranslated region (3′-UTR) containing the putative miR-593 or miR-511 binding site was amplified by PCR using the following primers: forward, 5′-ACCAAGCTTGTACCA CCAAGGTGATCCC-3′ and reverse, 5′-ACCACTAGTGCA GGCAGCTCCACCGAGGCATC-3′. A mutant 3′-UTR of *PROP1* was synthesized by PCR, whose sequence contained 5′-TCGTGAA***T***A***T***ACAAGAAAATG-3′ or 5′-GCTACTGG AA***G***A***G***A***C***AG GGCAAG-3′ (the letters in italic and bold font indicate nucleotides which are mutated) and cloned downstream of the luciferase gene in the pMIR-report luciferase vector (Ambion, Inc.). This construct, named *PROP1*-3′-UTR or *PROP1*-3′-UTR-Mut was used for the transfection of HEK293T cell lines. HEK293T cells were cultured in 24-well plates. In each well, 10 ng of *Renilla* luciferase, phRL-TK vector (Promega, Madison, WI, USA) were co-transfected to normalize for transfection effciency. A total of 500 ng of REPORT, *PROP1-*3′-UTR or *PROP1*-3′-UTR-Mut together with 10 nM miR-593 or miR-511 or the negative control was also co-transfected into the cells in 24-well plates. Transfection was carried out using Lipofectamine 2000 and Opti-MEM I reduced serum medium (Life Technologies, Carlsbad, CA, USA) in a final volume of 0.5 ml. The transfection of the same combinations of plasmid and RNAs as repeated 3 times. After 48 h, the cells were harvested with 100 *μ*l PLB reagent (Promega) and 20 *μ*l cell lysates prepared in Reporter Lysis Buffer (Promega), Firefly luciferase activity was measured for each well using the Dual luciferase assay kit (Promega) with an analytical luminometer (TD-20/20; Turner Designs, Sunnyvale, CA, USA) according to the manufacturer’s instructions. Briefly, a 10% volume of cell lysate (20 *μ*l) was added to 100 *μ*l of LAR II, and then the reaction was terminated by the addition of 100 *μ*l Stop and Glo^®^ Reagent. Normalized relative luciferase activity (RLA) was calculated using the following formula: RLA = (Firefly luciferase)/(*Renilla* luciferase).

### Statistical analysis

The differences between groups were estimated using the Pearson χ^2^ test, Student’s t-test and ANOVA test. Receiver operating characteristic (ROC) curves and the area under the curve (AUC) calculations were performed to determine the threshold value of the children with CPHD and the normal controls. SPSS 15.0 software (SPSS Inc., Chicago, IL, USA) was used for all statistical analyses, and a value of P<0.05 was considered to indicate a statistically significant difference. Data are expressed as the means ± standard deviation from at least 3 separate experiments.

## Results

### Global serum miRNA profiling and data analysis in children with CPHD

**Figure 1:**
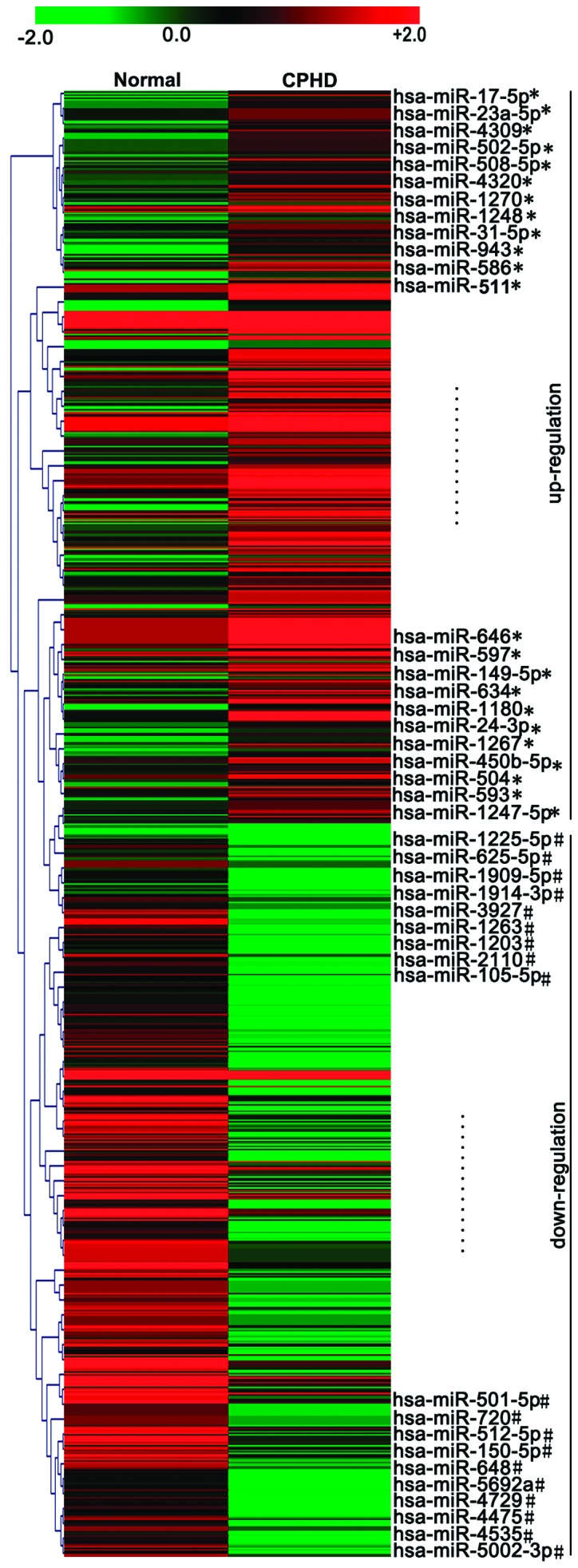
Heatmap and hierarchical clustering of miRNAs in serum of children with combined pituitary hormone deficiency (CPHD). The heatmap represents the results of the two-way hierarchical clustering of miRNAs in the serum of children with CPHD. Each row represents an miRNA, and each column represents samples tested. The clustering is represented for the miRNAs and samples on top. Red color represents miRNAs with an expression level above the mean value, and green color represents miRNAs with an expression level below the mean value. The 23 upregulated and 19 downregulated miRNAs identified are marked by an asterisk (*) or a hash (#) symbol, respectively.

**Figure 2:**
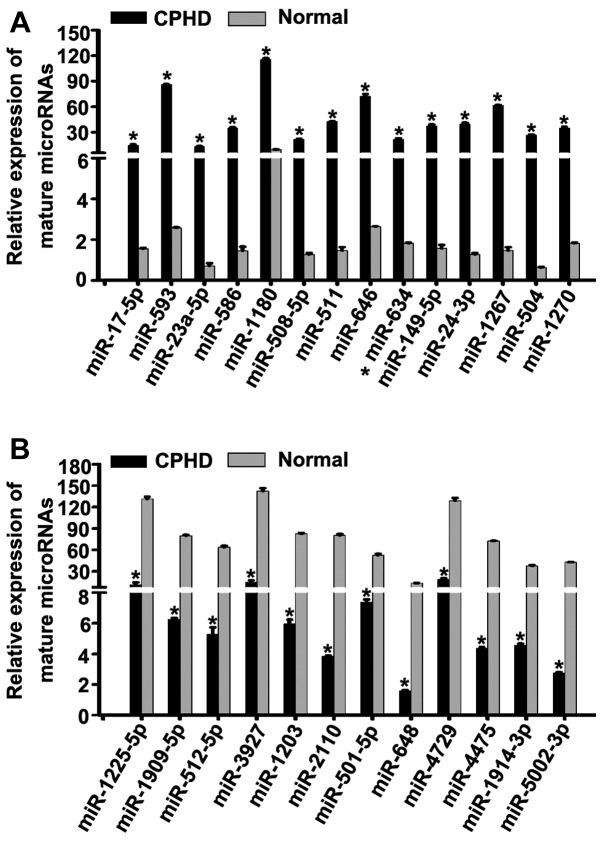
Identification of candidate miRNAs. (A) We detected the expression levels of 23 upregulated miNAs in children with combined pituitary hormone deficiency (CPHD) and normal (healthy) controls by RT-qPCR. The expression levels of miR-17-5p, miR-593, miR-23a-5p, miR-586, miR-1180, miR-508-5p, miR-511, miR-646, miR-634, miR-149-5p, miR-24-3p, miR-1267, miR-504 and miR-1270 were upregulated compared to the normal controls. (B) We examined the expression levels of 19 downregulated miRNAs in children with CPHD and normal controls by RT-qPCR. The expression levels of miR-1225-5p, miR-1909-5p, miR-512-5p, miR-3927, miR-1203, miR-2110, miR-501-5p, miR-648, miR-4729, miR-4475, miR-1914-3p and miR-5002-5p were downregulated compared to the normal controls. ^*^P<0.05. Each bar represents the mean value ± standard deviation from 3 independent experiments.

miRNA expression profiles from the serum samples of 7 children with CPHD and 7 normal controls were analyzed by microarray analysis. Hierarchical clustering analyses of the overall expression profile divided the samples into 2 groups: children with CPHD and normal (healthy) controls (Fig. 1). The threshold set for up- and downregulated miRNAs was a fold change ≥6.0 and a P-value ≤0.05. Our results revealed 23 upregulated and 19 downregulated miRNAs with abnormal expression levels in children with CPHD compared with the normal controls (Fig. 1 and Table I). The expression levels of miR-17-5p, miR-593, miR-23a-5p, miR-586, miR-1180, miR-508-5p, miR-511, miR-646, miR-634, miR-149-5p, miR-24-3p, miR-1267, miR-504 and miR-1270 were upregulated (Fig. 2A) (P<0.05). However, the expression levels of miR-1225-5p, miR-1909-5p, miR-512-5p, miR-3927, miR-1203, miR-2110, miR-501-5p, miR-648, miR-4729, miR-4475, miR-1914-3p and miR-5002-5p were downregulated (Fig. 2B) (P<0.05) in the children with CPHD compared with the normal controls.

### Prediction and identification of the candidate miRNAs, miR-593 and miR-511, which directly target the PROP1 gene

**Figure 3:**
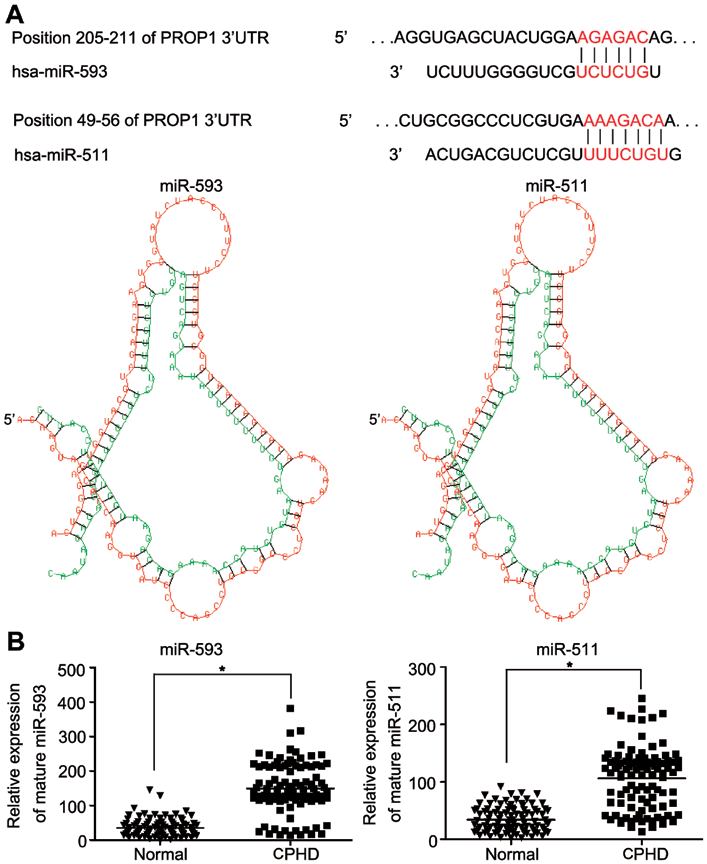
Candidate miRNAs, miR-593 and miR-511, were upregulated in children with combined pituitary hormone deficiency (CPHD) as shown by RT-qPCR. (A) We used several computational methods to predict the potential miR-593 and miR-511 targets using the microrna.org, TargetScan and RNAhybrid database online searching programs; only matched nucleotides with miRNA seed sequences are indicated with vertical lines. We identified that the *PROP1* gene was a possible was a target of miR-593 and miR-511. (B) We examined the expression levels of miR-593 and miR-511 in the 103 children with CPHD and the 103 normal controls by RT-qPCR. The mean expression levels of miR-593 and miR-511 were upregulated compared with the normal controls. ^*^P<0.05. Each bar represents the mean value ± standard deviation from 3 independent experiments.

Among the targets predicted by the microrna.org, TargetScan databases and RNAhybrid database online search programs, we identified the *PROP1* gene as a possible target of miR-593 and miR-511 (Fig. 3A). The results of the database search suggested the association of miR-593 and miR-511 with the *PROP1* gene. The expression levels of miR-593 and miR-511 in the 103 children with CPHD and the normal controls were examined. The mean expression level of miR-593 was 149.06±72.34 and that of miR-511 was 106.18±54.08. The mean expression levels of miR-593 (149.06±72.34 vs. 34.89±24.61) and miR-511 (106.18±54.08 vs. 34.21±21.53) were upregulated compared to the normal controls (Fig. 3B; P<0.05).

**Figure 4:**
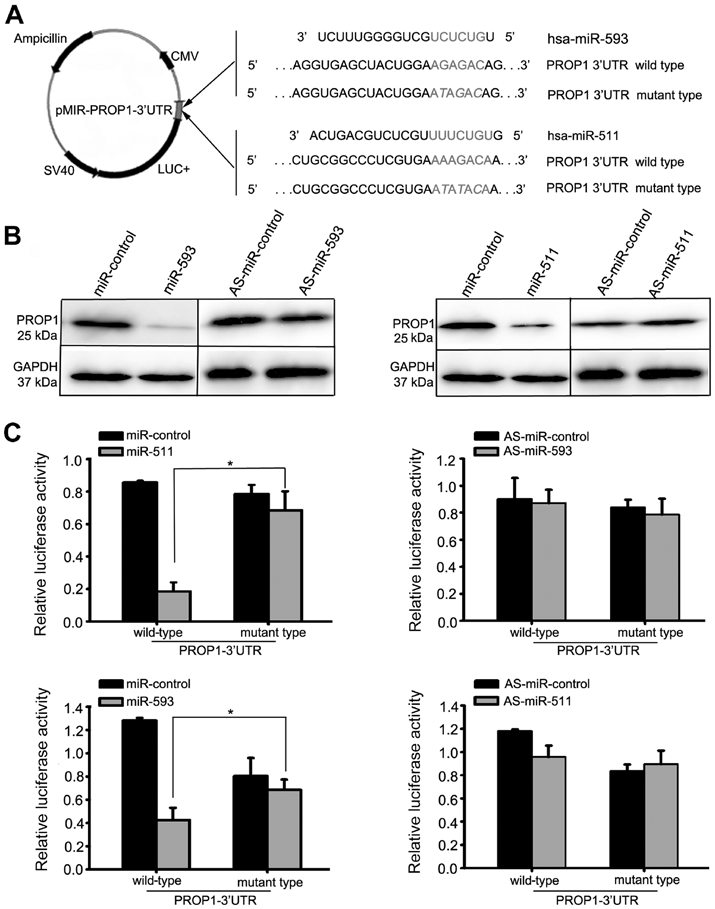
miR-593 and miR-511 target the 3′-untranslated regions (3′-UTR) of the *PROP1* gene and decrease PROP1 expression. (A) Representative nucleotide sequence matches between possible target sequences and miRNAs. The miR-593 seed sequence (UCUCUG) and miR-511 seed sequence (UUUCUGU) is shown (gray and italic nucleotides). Schematic graph of the 3′-UTR binding site for miR-593 or miR-511. *PROP1*-3′-UTR-wild-type or *PROP1*-3′-UT-mutant type was inserted downstream of the luciferase of pMIR-reporter vector. (B) At 48 h after the transfection of miR-593 precursor, miR-511 precursor, miR-593 inhibitor, miR-511 inhibitor or control oligonucleotides into the HEK293T cells, the PROP1 protein level was significantly reduced in the cells transfected with miR-593 precursor or miR-511 precursor, as shown by western blot analysis. By contrast, transfection with miR-593 inhibitor or miR-511 inhibitor did not affect the protein expression of PROP1. (C) We assessed the luciferase activity by co-transfecting the luciferase reporter vector bearing the 3′-UTR of the *PROP1* gene with the miR-593 precursor, miR-511 precursor, miR-593 inhibitor, miR-511 inhibitor or control oligonucleotides. Luciferase activity of reporter plasmid with wild-type 3′-UTR of the *PROP1* gene was markedly decreased in the cells transfected with miR-593 precursor and miR-511 precursor, compared with the luciferase activity of the reporter plasmid with mutant 3′-UTR of *PROP1*. Each bar represents the mean values ± SD from 3 independent experiments (^*^P<0.05).

To verify the direct interaction between miR-593 and miR-511 and the 3′-UTR of the *PROP1* gene, we cloned the 3′-UTR region that was predicted to interact with miR-593 and miR-511 into a luciferase reporter vector (Fig. 4A). The HEK293T cells were transfected with the miR-593 precursor, miR-511 precursor, miR-593 inhibitor, miR-511 inhibitor or control oligonucleotides. As is shown in Fig. 4B, the upregulation of miR-593 and miR-511 inhibited PROP1 protein expression by approximately 87%. The luciferase activity of the reporter plasmid with the wild-type 3′-UTR of the *PROP1* gene was markedly decreased in the cells transfected with the miR-593 precursor and miR-511 precursor compared to the luciferase activity of the reporter plasmid with the mutant 3′-UTR of the *PROP1* gene (Fig. 4C; P<0.05). Conversely, the luciferase activity of the reporter plasmid was not affected following transfection with miR-593 inhibitor and miR-511 inhibitor compared to the anti-sense (AS)-miR-control (Fig. 4C) (P<0.05).

### miR-593 and miR-511 as serum biomarkers for children with CPHD

**Figure 5:**
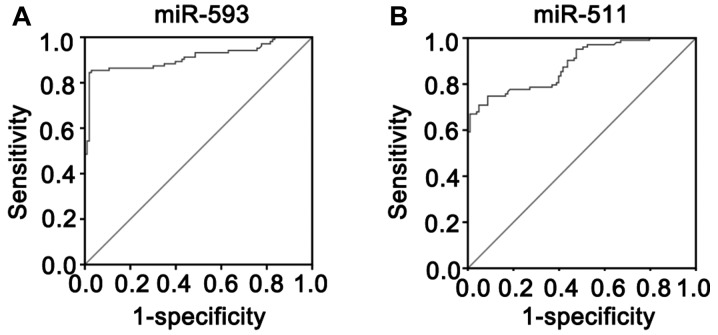
miR-593 and miR-511 may serve as serum biomarkers for children with combined pituitary hormone deficiency (CPHD). (A) The ROC curve of the serum of the 103 children with CPHD and the 103 normal controls for miR-593. The AUC was 0.912±0.02 for miR-593, with a sensitivity and specificity of 82.54 and 98.15%, respectively. (B) The ROC curve of the serum of the 103 children with CPHD and the 103 normal controls for miR-511. The AUC was 0.785±0.023 for miR-511, with a sensitivity and specificity of 84.86 and 91.36%, respectively.

The expression of miR-593 and miR-511 in the children with CPHD was significantly increased compared with the normal controls. The AUC was 0.912±0.020 for miR-593 and was significantly higher than that of the null hypothesis (true area was 0.5) (Fig. 5A; P<0.01). The AUC was 0.785±0.023 for miR-511 and was significantly higher than that of the null hypothesis (true area was 0.5) (Fig. 5B; P<0.01). According to Youden’s index, the optimal operating point of the expression level of miR-593 and miR-511 in the serum was 108.27 and 63.43, respectively. At this cut-off point, the sensitivity was 82.54 and 84.86%, and the specificity was 98.15 and 91.36% for miR-593 and miR-511, respectively.

## Discussion

The pituitary gland produces hormones that play important roles in the development and the homeostasis of the body. A deficiency of two or more of these pituitary hormones, known as CPHD, may present in infants or children due to an unknown etiology and is considered congenital or idiopathic. To date, to the best of our knowledge, there is no study available on miRNAs in CPHD. miRNAs are a class of small non-coding RNAs and have been discovered in animals and plants. miRNAs are 19–22 nucleotide non-coding RNAs and are able to bind complementary sequences in the 3′-UTR of target mRNAs to induce their degradation or translational repression (24–26). They are encoded by genes that are presumably transcribed into single or clustered primary transcripts, which are processed and produce mature miRNAs. They function as regulators of disease initiation, progression and metastasis. miRNAs are a novel class of regulatory molecules with the ability to control gene expression at the post-transcriptional level. They appear decrease protein expression by blocking the translation of mRNAs into proteins. Therefore, the identification of CPHD-specific miRNAs is critical for understanding their role in the pathophysiological basis of CPHD and may prove useful for finding novel therapeutic methods. Intriguingly, it has been suggested that miRNAs are present in human peripheral blood in a consistent, reproducible and stable manner (19). More importantly, a series of studies have demonstrated that at least in some pathological conditions, such as cancer, heart failure and liver damage, circulating miRNAs may in part reflect tissue damage (13,14,27,28). This discovery opens up the possibility of using miRNAs as non-invasive biomarkers for CPHD.

Hitherto, there data on the association between miRNAs and CPHD are limited. Microarray analysis is a powerful technology that is able to perform genome-wide analysis in one experiment. miRNA expression profiles can characterize miRNAs that are differentially regulated under different experimental conditions. Thus, in this study, we used a global miRNA microarray to identify specific miRNAs in CPHD. Our results revealed 23 upregulated and 19 downregulated miRNAs that were abnormally expressed in children with CPHD compared with the normal controls. The expression levels of miR-17-5p, miR-593, miR-23a-5p, miR-586, miR-1180, miR-508-5p, miR-511, miR-646, miR-634, miR-149-5p, miR-24-3p, miR-1267, miR-504 and miR-1270 were upregulated. The expression levels of miR-1225-5p, miR-1909-5p, miR-512-5p, miR-3927, miR-1203, miR-2110, miR-501-5p, miR-648, miR-4729, miR-4475, miR-1914-3p and miR-5002-5p were downregulated in the children with CPHD. These data indicate that the characteristics of serum miRNA expression are associated with children with CPHD and provide a valuable repertoire that can be used to discover circulating miRNA-based biomarkers for the diagnosis of CPHD. To better understand the role of specific miRNAs in the diagnosis of CPHD, the microrna.org, TargetScan and RNAhybrid database online searching programs were used to predict putative targets. We identified that the *PROP1* gene was a possible target of miR-593 and miR-511; further experimental procedures were carried out to validate these findings. Our results revealed that the expression levels of miR-593 or miR-511 in the serum of children with CPHD were upregulated compared with the normal controls. miR-593 and miR-511 directly targeted the *PROP1* gene and this was confirmed by western blot analysis and luciferase activity assay following the transfection of HEK293T cells with miR-593 precursor, miR-511 precursor, miR-593 inhibitor, miR-511 inhibitor or control oligonucleotides. The above findings support the hypothesis that miRNAs may be involved in the development of CPHD. In clinical practice, the tissue in children with CPHD is not readily accessible. The ROC curve and the AUC for the expression levels of the serum of the 103 children with CPHD and the 103 normal controls were calculated. The AUC was 0.912±0.020 for miR-593 and the AUC was 0.785±0.023 for miR-511. Thus, the expression levels of miR-593 and miR-511 in serum may serve as a molecular marker for children with CPHD. To identify an optimal cut-off point to detect children with CPHD, Youden’s index was used in this study. According to Youden’s index, the optimal operating point of the blood expression level of miR-593 and miR-511 was 108.27 and 63.43, respectively. At this cut-off point, the sensitivity was 82.54 and 84.86%, specificity was 98.15 and 91.36% for miR-593 and miR-511, respectively. Taken together, these data suggest that miR-593 and miR-511 directly target the *PROP1* gene and may serve as serum biomarkers for children with CPHD.

In conclusion, in the present study, we found that the levels of miR-593 and miR-511 in the serum of children with CPHD were significantly increased and that these miRNAs directly targeted the *PROP1* gene. This suggests that circulating levels of miR-593 or miR-511 may serve as novel biomarkers for the clinical diagnosis of CPHD. Thus, miRNAs cannot be overlooked as a class of molecules that regulate biological functions and CPHD. Our data broaden the understanding of the functions of miRNAs in children with CPHD. Considering the small sample size used in the present study, investigations including a larger scale of patients are warranted. Additionally, further studies are required to reveal the exact time course of miR-593 and miR-511 in the serum of children with CPHD. Although circulating miRNA levels can be detected by real-time PCR, the underlying mechanisms responsible for the increased circulating miRNA levels and whether they have pathophysiological functions in CPHD require further investigation.

## Figures and Tables

**Table I:** Differentially expressed miRNAs in children with CPHD with at least a 6-fold change in epxression selected by microarray data analysis.

No.	Name	Corrected P-value	Fold change	Regulation
1	hsa-miR-17-5p	0.00038	9.737702	Up
2	hsa-miR-23a-5p	0.00004531	7.1713095	Up
3	hsa-miR-4309	0.000814	13.075409	Up
4	hsa-miR-502-5p	0.0000028	12.420118	Up
5	hsa-miR-508-5p	0.000018	8.289489	Up
6	hsa-miR-4320	0.000029	7.698423	Up
7	hsa-miR-1270	0.00000478	7.7110386	Up
8	hsa-miR-1248	0.001356	16.126984	Up
9	hsa-miR-31-5p	0.000029	8.6424675	Up
10	hsa-miR-943	0.00645	7.1091013	Up
11	hsa-miR-586	0.000781	9.900925	Up
12	hsa-miR-511	0.00000569	13.058901	Up
13	hsa-miR-646	0.0000056	6.3428288	Up
14	hsa-miR-597	0.0000848	8.018958	Up
15	hsa-miR-149-5p	0.00264	8.6524935	Up
16	hsa-miR-634	0.000157	10.592684	Up
17	hsa-miR-1180	0.000457	11.971244	Up
18	hsa-miR-24-3p	0.0000063	12.153815	Up
19	hsa-miR-1267	0.000487	6.773687	Up
20	hsa-miR-450b-5p	0.000158	8.018958	Up
21	hsa-miR-504	0.0000056	6.3428288	Up
22	hsa-miR-593	0.000158	8.018958	Up
23	hsa-miR-1247-5p	0.0000651	6.3428288	Up
1	hsa-miR-1225-5p	0.000024	−6.7412977	Down
2	hsa-miR-625-5p	0.000467	−12.856266	Down
3	hsa-miR-1909-5p	0.000267	−11.81031	Down
4	hsa-miR-1914-3p	0.000984	−8.549995	Down
5	hsa-miR-3927	0.000289	−14.380777	Down
6	hsa-miR-1263	0.000451	−9.568466	Down
7	hsa-miR-1203	0.000167	−10.962637	Down
8	hsa-miR-2110	0.000287	−11.463881	Down
9	hsa-miR-105-5p	0.0000671	−6.78255	Down
10	hsa-miR-501-5p	0.0000205	−8.172279	Down
11	hsa-miR-720	0.000091	−10.1659565	Down
12	hsa-miR-512-5p	0.00036	−17.136076	Down
13	hsa-miR-150-5p	0.000497	−8.718422	Down
14	hsa-miR-648	−7.745552	−6.179926	Down
15	hsa-miR-5692	−10.1659565	−7.8253	Down
16	hsa-miR-4729	−7.745552	−13.084776	Down
17	hsa-miR-4475	−6.1597967	−9.571002	Down
18	hsa-miR-4535	−7.383092	−7.945036	Down
19	hsa-miR-5002-3p	−5.559328	−10.245269	Down

The threshold set for up- and downregulated microRNAs was a fold change ≥6.0 and a P-value ≤0.05. Our results found 23 upregulated and 19 downregulated microRNA abnormally expressed in children with CPHD compared with the normal (healthy) controls. miRNAs, microRNAs; CPHD, combined pituitary hormone deficiency.
