# [Corrigendum] Circulating microRNA profiles and the identification of miR-593 and miR-511 which directly target the PROP1 gene in children with combined pituitary hormone deficiency

**DOI:** 10.3892/ijmm.2026.5941

**Published:** 2026-07-29

**Authors:** Yanyan Hu, Qian Wang, Zengmin Wang, Fengxue Wang, Xiaobo Guo, Guimei Li

Int J Mol Med 35: 358-366, 2015; DOI: 10.3892/ijmm.2014.2016

Following the publication of this paper, it was drawn to the Editor's attention by a concerned reader that, regarding the western blot data shown in Fig. 4B on p. 364, the GAPDH blots were shown to be the same for the experiments performed with miR-593 and miR-511, which was not anticipated, given the different experimental conditions. Secondly, one set of the control GAPDH blots, and also potentially the PROP1 blots for the experiments performed with miR-593, had apparently already been published in an article in the journal *PLoS One*, which held one of the authors listed above (Xiaobo Guo) in common. Thirdly, the blots shown for the AS-miR control and AS-miR-miR-593 blots (left-hand gels) bore a strong similarity to those featured for the AS-miR-control and AS-miR-511 blots, albeit with vertical resizing of one set of the protein bands.

After contacting the authors about this issue, they have replied to say that unintentional errors occurred during the assembly of the data in this figure, involving the use of the loading control images and the presentation of the PROP1 bands. Given the period of time that has elapsed since this paper was published, the authors no longer had access to the original data; therefore, they have repeated these experiments. The results of the new western blotting experiments were broadly similar to those of the original experiments, and the revised version of Fig. 4, now featuring the new western blotting data for Fig. 4B, is shown on the next page. The authors confirm that the errors made in assembling the data in Fig. 4 did not have a significant impact on either the results or the conclusions reported in this study, and all the authors agree with the publication of this Corrigendum. The authors are grateful to the Editor of *International Journal of Molecular Medicine* for allowing them the opportunity to publish this Corrigendum; furthermore, they apologize to the readership of the Journal for any inconvenience caused.

## Figures and Tables

**Figure 4:**
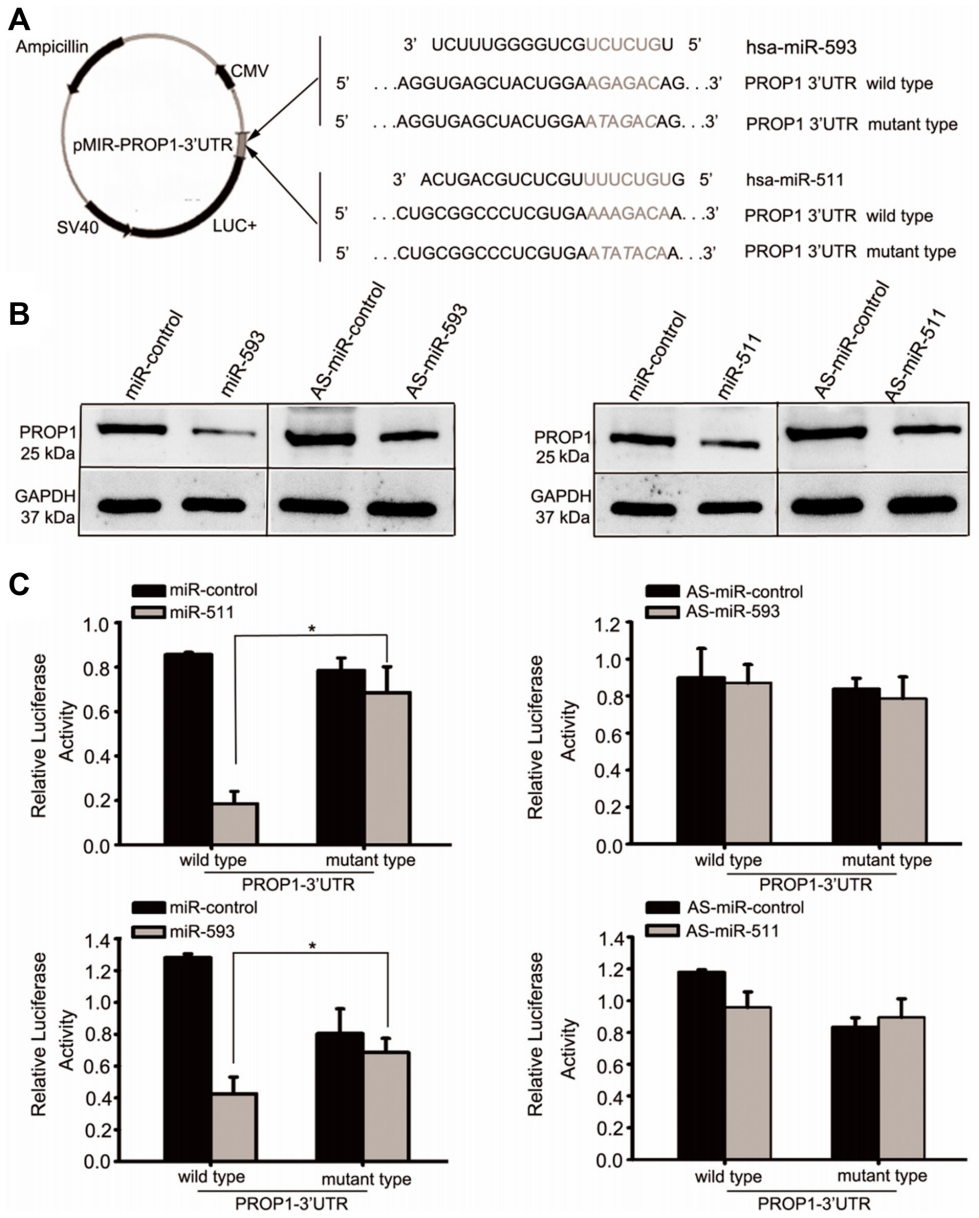
miR-593 and miR-511 target the 3'-untranslated regions (3'-UTR) of the PROP1 gene and decrease PROP1 expression. (A) Representative nucleotide sequence matches between possible target sequences and miRNAs. The miR-593 seed sequence (UCUCUG) and miR-511 seed sequence (UUUCUGU) is shown (gray and italic nucleotides). Schematic graph of the 3'-UTR binding site for miR-593 or miR-511. PROP1-3'-UTR-wild-type or PROP1-3'-UT-mutant type was inserted downstream of the luciferase of pMIR-reporter vector. (B) At 48 h after the transfection of miR-593 precursor, miR-511 precursor, miR-593 inhibitor, miR-511 inhibitor or control oligonucleotides into the HEK293T cells, the PROP1 protein level was significantly reduced in the cells transfected with miR-593 precursor or miR-511 precursor, as shown by western blot analysis. By contrast, transfection with miR-593 inhibitor or miR-511 inhibitor did not affect the protein expression of PROP1. (C) We assessed the luciferase activity by co-transfecting the luciferase reporter vector bearing the 3'-UTR of the PROP1 gene with the miR-593 precursor, miR-511 precursor, miR-593 inhibitor, miR-511 inhibitor or control oligonucleotides. Luciferase activity of reporter plasmid with wild-type 3'-UTR of the PROP1 gene was markedly decreased in the cells transfected with miR-593 precursor and miR-511 precursor, compared with the luciferase activity of the reporter plasmid with mutant 3'-UTR of PROP1. Each bar represents the mean values ± SD from 3 independent experiments (^*^P<0.05).

